# Mental health literacy among family nurses in Poland: a cross-sectional study

**DOI:** 10.3389/fpubh.2026.1942327

**Published:** 2026-09-30

**Authors:** Mariola Pietrzak, Anna Mierzejewska, Monika Tomaszewska, Grzegorz Han, Barbara Ślusarska, Grzegorz Józef Nowicki

**Affiliations:** 1 Department of Development of Nursing, Social and Medical Sciences, Medical University of Warsaw, Warsaw, Poland; 2 Faculty of Health Sciences, Medical University of Warsaw, Warsaw, Poland; 3 Institute of Nursing, Warsaw University of Medical Sciences, Warsaw, Poland; 4 Optima Medycyna S.A. Healthcare Center in Prudnik, Prudnik, Poland; 5 Department of Family and Geriatric Nursing, Faculty of Health Sciences, Medical University of Lublin, Lublin, Poland

**Keywords:** family nursing, mental health literacy, mental health nursing, primary health care, workforce education, Poland, cross-sectional study

## Abstract

**Background:**

More than a billion people worldwide suffer from mental health disorders, and primary health care (PHC) is critical for early recognition and treatment of these medical conditions. Family nurses (FNs), as the patient's first point of contact with the healthcare system, are considered to play an important role in identifying mental health disorders. Mental health literacy (MHL) refers to the knowledge and beliefs that help with the recognition, treatment and prevention of mental disorders; yet, little is known about the level of MHL among family nurses in Poland. The aim of this study was to assess the level of MHL among family nurses in Poland and to identify sociodemographic and occupational factors that are independently linked with it.

**Methods:**

From February to May 2026, a total of 207 family nurses participated in a cross-sectional study that utilized the CAWI method and regionally stratified sampling of primary health care facilities. The Polish version of the Mental Health Literacy Scale (MHLS-PL) and the authors' original questionnaire on sociodemographic and occupational variables were employed.

**Results:**

The median normalized total score for MHLS-PL was 70.43%. The Knowledge of risk factors and causes and Knowledge of professional help available subscales had the lowest normalized scores (66.67% each), while the Recognition of disorders and Knowledge of how to seek mental health information subscales had the highest normalized scores (75% each). Factors independently associated with the total MHLS-PL score were: younger age, urban residence, having a family member or acquaintance with a mental disorder, and professional interaction with individuals with mental disorders. Analyses of individual subscales additionally identified educational level (master's degree or higher), shorter length of service, and personal mental health diagnosis as significant correlates for specific knowledge and attitude dimensions.

**Conclusion:**

The level of MHL among Polish nurses varied across subscales, with the most knowledge gaps being in the understanding of risk factors and the etiology of mental diseases. The negative relationship between age, length of service and MHL suggests potential target groups for future educational interventions, particularly experienced nurses and those in rural areas, which should be evaluated in subsequent research. The study results provide initial insights that can inform the planning of educational interventions and health policies, although further research is needed to evaluate their effectiveness and to address organizational and system-level factors that may influence the translation of MHL into clinical practice.

## 1 Introduction

Mental disorders are one of the greatest challenges to modern medicine and public health, impacting over one billion people globally (1, 2). It is estimated that about one-quarter of the global population will face a mental health crisis at some point in their lives, and the number of cases of depression, anxiety disorders, as well as other problems has increased drastically in the post-COVID-19 era (3, 4). According to World Health Organization (WHO) data, approximately 20% of all appointments at primary health care facilities concern mental health disorders (5), indicating the importance of this care in the early recognition, diagnosis and treatment of such medical conditions. Moreover, the global burden of mental diseases is gradually expanding, and the gap between the population's health requirements and the availability of specialized psychiatric care remains large, especially given the shortage of qualified specialists (6, 7). Therefore, primary health care and, notably, the nurses who work within this structure, play key roles in incorporating psychiatric care into ordinary clinical practice (8). For many years, the WHO and other international bodies have recommended the integration of psychiatric care with primary health care and have considered doing such as the best sustainable, cost-effective and scalable strategy for reducing the treatment gap (9). Family nurses are frequently the first healthcare professionals to encounter persons suffering from mental health disorders, putting them in a strategic position to facilitate early detection, prompt intervention and referral to specialists (10).

Mental health literacy (MHL) is a crucial aspect in assessing the effectiveness, especially amongst healthcare professionals practicing in primary health care, of healthcare systems in battling the increasing prevalence of mental disorders in the population. This concept, which was introduced by Jorm et al. (11), covers knowledge and beliefs about mental disorders that help in their recognition, treatment or prevention. MHL encompasses, among other issues, the ability to recognize symptoms of mental diseases, knowledge of risk and protective factors, knowledge of various treatment methods, the capacity to obtain accurate information, as well as of attitudes that support or reject seeking professional help. These dimensions are interconnected yet heterogeneous as their distribution among the population might vary greatly, emphasizing the need for a comprehensive assessment.

While the concept of MHL originated in studies of the general public, its relevance to healthcare professionals has gained increasing recognition (12). The Mental Health Literacy Scale (MHLS), originally developed by O'Connor and Casey for the general population, has been subsequently adapted and validated for use among primary healthcare workers, including nurses (13). Studies have confirmed the construct validity and strong internal consistency of the revised MHLS in primary care settings (14). Its application in professional groups is further supported by evidence that healthcare practitioners score significantly higher on the MHLS than the general population, indicating the scale's capacity to detect relevant differences in MHL (15). Nevertheless, as a measure of general MHL, the MHLS does not fully capture profession-specific competencies, such as advanced clinical communication, suicide risk assessment, or the practical application of mental health knowledge in patient care-aspects that are essential to nursing practice (16). This limitation has been acknowledged in previous research, which noted a gap between professional researchers' and clinical workers' knowledge and attitudes related to mental health (12). Therefore, while the MHLS provides a valuable foundation for assessing baseline MHL among family nurses, its results should be interpreted with awareness of these limitations, which are addressed further in the discussion.

While the present study focuses on individual-level MHL among family nurses, contemporary health literacy frameworks increasingly emphasize that individual competencies are shaped by, and interact with, the organizational and system-level environment in which healthcare is delivered (13, 17). Organizational Health Literacy (OHL) refers to the degree to which healthcare organizations enable individuals to find, understand, and use health information and services to inform health-related decisions and actions (18, 19). This perspective marks a shift from viewing health literacy as solely an individual trait to recognizing it as a shared responsibility of healthcare systems and organizations, as organizational structures and processes can either hinder or support individuals' ability to navigate and benefit from care (20, 21). Primary care settings, including those where family nurses practice, are particularly relevant for OHL, as they represent the first and most frequent point of contact for patients (22, 23). Studies have shown that strengthening OHL in primary care—through improved access, communication, navigation, and staff development—can enhance both patient outcomes and the effectiveness of healthcare delivery (24). Although the present study did not directly assess OHL, acknowledging this organizational dimension provides a complementary perspective for interpreting the findings. Future research could explore how organizational factors, such as training policies, interprofessional collaboration, and institutional support for mental health care, may influence or moderate the relationship between nurses' individual MHL and their clinical performance. This aligns with broader discussions on the changing role of health professionals in health-system reform, particularly in the post-pandemic era, where the contributions of various professional groups are being re-evaluated to strengthen primary care and community-based services (25).

While OHL focuses on the internal capacity of healthcare organizations, contemporary health literacy frameworks increasingly emphasize that sustainable improvements require extending beyond organizational boundaries. Intersectoral collaboration - understood as cooperative relationships between public and private organizations from different sectors - has been recognized as a critical driver for addressing complex health and social challenges (26, 27). This approach aligns with the principles of co-design, which actively involves diverse stakeholders, including patients, healthcare providers, and community representatives, in the collaborative development of health and social care services (28). As demonstrated by Giusti and Persiani (29) in the context of Alzheimer's disease, intersectoral collaboration and co-design can facilitate the integration of non-pharmacological interventions, such as art therapy, into care pathways by engaging public and private organizations across sectors. Translating this perspective to primary mental health care, effective MHL interventions for family nurses may similarly benefit from collaborative approaches that extend beyond individual professional education. Partnerships between primary care providers, mental health specialists, social services, educational institutions, and community organizations could enable the co-creation of training programs that are responsive to local needs, culturally appropriate, and sustainably implemented (22, 24). Although the present study did not directly assess intersectoral collaboration or co-design processes, acknowledging this broader context provides a complementary lens for interpreting the findings and suggests that future research and practice should consider how multi-stakeholder partnerships can enhance the translation of MHL into effective, person-centered care.

Recent study has considerably improved our understanding of MHL among nurses and other healthcare professionals. Jansen and Morifi (30) examined the ability of South African family nurses to recognize mental disorders, finding that while depression and anxiety disorders were diagnosed reasonably well, the rate of diagnosis for post-traumatic stress disorder (PTSD) remained notably low. A commentary on this study stressed the importance of targeted mental health literacy training for family nurses in enhancing early detection of mental disorders (31). Güden et al. (32) carried out a randomized controlled trial to assess a brief online mental health literacy training program for primary health care professionals in Turkey and discovered that such intervention significantly improved mental health literacy levels while lowering perceptions of the risks related to a mental disease. In a systematic review and meta-analysis of interventions aiming at reducing mental health stigma among PHC professionals, Zhamaliyeva et al. (33) discovered that direct-contact interventions and multi-component interventions had stronger and longer-lasting effects. Four overarching aspects regarding the role of nurses in psychiatric care in primary health care were identified in a qualitative metasynthesis by Rafi et al. (34): lowering stigma, removing structural barriers, implementing adaptive psychosocial interventions, as well as directing interdisciplinary, collaboration-based care. Medeiros et al. (2), in turn, stated that, while family nurses perceive mental health practices to be within their scope of practice and perform them, many do not feel fully competent to do so. A Spanish intervention study conducted by nurses within the framework of the collaboration between universities, schools and the community showed that educational intervention significantly improved the MHL level, attitudes, as well as willingness to seek help among all study groups, including school-age youth and future health educators. The above-mentioned results unequivocally identify nurses as crucial educators in the promotion of mental health in schools (35).

Despite an increasing amount of research on MHL, studies have predominantly focused on nursing students (36), the general population (37), or nurses in hospital settings (38), leaving a significant knowledge gap regarding MHL among family nurses in primary health care (39). This is particularly concerning because family nurses, as the patient's first point of contact with the healthcare system, are considered to have a potentially important role in early recognition, monitoring, and psychoeducation for patients with mental health problems. However, the determinants of MHL among family nurses in Central and Eastern Europe remain largely unexplored, with Poland being a notable example. This gap is especially critical given that available data show considerable MHL deficits among Poland's general population: nearly half of all adults are unable to recognize symptoms of mental disorders, and one-third are unsure where or when to seek help (40, 41). Addressing these deficits at the community level requires competent family nurses; yet, no study to date has comprehensively assessed MHL among Polish family nurses using a validated instrument. Therefore, the present study is needed to establish a baseline understanding of MHL levels and their associated factors in this under-researched professional group, which can subsequently inform targeted educational interventions and health policy.

## 2 Materials and methods

### 2.1 Study design and participants

The cross-sectional study was conducted utilizing computer-assisted web interviewing (CAWI) between February and May 2026. The CAWI method allowed the authors to reach a broad range of respondents across the country, minimized the impact of geographical barriers resulting from dispersed primary health care facilities, and at the same time ensured anonymity and convenience in completing the questionnaire. In order to ensure that the survey had the broadest possible scope and included respondents from all regions of the country, an email invitation to participate in the study, along with a link to the questionnaire, was sent to the managers of primary health care facilities in all Polish provinces. The facility managers were then requested to inform all family nurses practicing at the subordinate institutions about the study.

The primary health care facilities were chosen for the study purposes utilizing stratified sampling, with the number of residents in each province serving as the stratification criterion, guided by the principle of proportionality to population size. In 10 provinces with populations exceeding two million, four primary health care facilities were chosen randomly from each province - two in urban areas and two in rural areas. The remaining six provinces, each with a population of less than two million, were reduced to two facilities, i.e., one for each category of location.

This approach allowed the authors to obtain a regionally stratified sample. In order to increase the number of responses and limit the risk of non-response bias, reminders were provided to the primary care facility manager on the first and tenth day of each month during the study period, via the same communication means used during the recruitment procedure. The response rate could not be calculated precisely, as the invitation to participate was distributed by facility managers rather than sent directly to individual nurses, and the number of nurses who received the invitation could not be determined. Nevertheless, reminders were sent to facility managers on the first and tenth day of each month during the study period to maximize participation.

The inclusion criteria were as follows: (1) holding a valid nursing license; (2) practicing as a family nurse at a primary health care facility for at least 1 month before the commencement of the study; and (3) giving informed consent to participate in the study. The exclusion criteria included: (1) practicing as a nurse outside of the primary health care sector; (2) practicing as a family nurse for less than 1 month before the commencement of the study; (3) failing to provide informed consent to participate in the study; and (4) incomplete completion of the survey questionnaire. All inclusion criteria were verified based on respondents' self-reported answers to the corresponding questions in the survey questionnaire, including confirmation of holding a valid nursing license, length of practice as a family nurse (at least 1 month), and provision of informed consent. Incomplete or non-confirmatory responses resulted in exclusion from the study.

Following the completion of the data collection process, the responses were subjected to a multi-stage data quality control in order to eliminate errors that could distort the final results (Figure 1). This procedure consisted of three stages.

**Figure 1:**
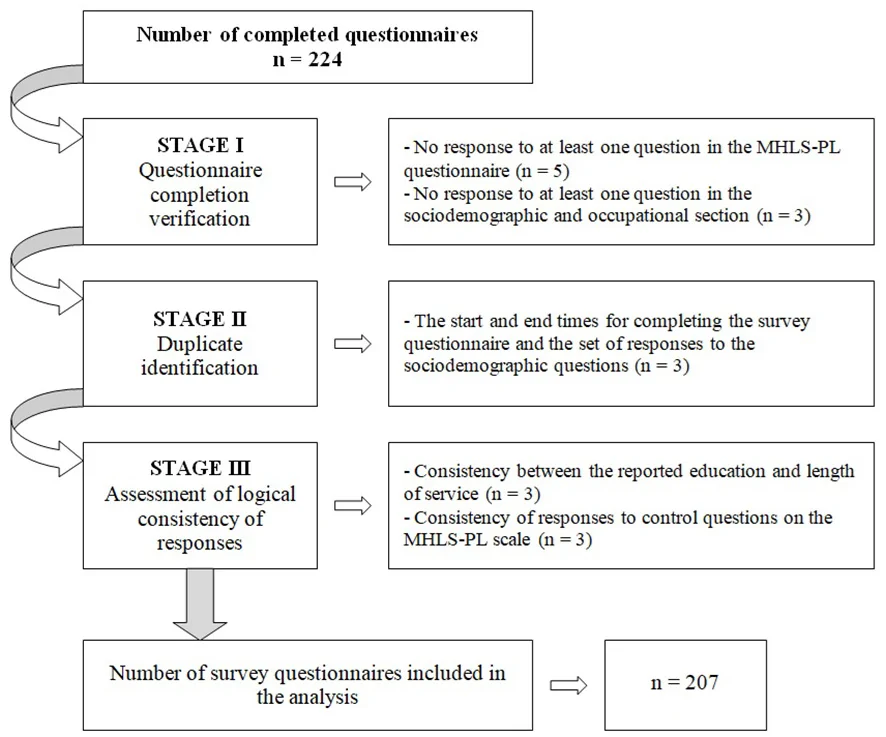
Flowchart of the data quality control process.

#### 2.1.1 Stage I: questionnaire completion verification

The first stage involved assessing the extent to which each item on the MHLS-PL questionnaire had been completed. Failure to respond to at least one question in the MHLS-PL questionnaire or one question in the section concerning sociodemographic and occupational characteristics resulted in exclusion from further analysis.

#### 2.1.2 Stage II: duplicate identification

In order to rule out the possibility of completing the survey questionnaire multiple times by the same respondent, the following indicators were examined: the timestamp (i.e., the start and end times of completing the survey questionnaire) in connection with the IP address of the device on which the survey questionnaire was completed, as well as the set of responses to sociodemographic questions (age, gender, length of service). If the completion times were similar (a difference of less than 5 min) and at least three of the six responses to sociodemographic questions were identical, the questionnaires were considered duplicates and eliminated from the study, leaving only one completed questionnaire. The threshold of a completion-time difference of less than 5 min was chosen to identify submissions that were too rapid to reflect genuine responses, while the criterion of at least three identical sociodemographic responses was applied to minimize the risk of falsely identifying unrelated submissions as duplicates. Although IP addresses were collected solely for the purpose of identifying potential duplicate submissions, they were not linked to the final data set and were deleted immediately after the duplicate-identification process. This approach ensured that the anonymity of respondents was preserved throughout the study.

#### 2.1.3 Stage III: assessment of logical consistency

The subsequent stage of the quality control process involved examining the logical consistency of the responses provided. Individual responses were assessed to determine whether they were consistent with one another and if there were any noticeable inconsistencies that could suggest that the survey questionnaire was filled out randomly or carelessly. The analysis involved the examination of the consistency of reported education and length of service, as well as the consistency of responses to control questions on the MHLS-PL scale.

After applying the exclusion criteria listed above and performing a quality control, a total of 207 correctly completed survey questionnaires were included in the study.

### 2.2 Questionnaire

To achieve the objectives of the study, a structured questionnaire was developed, consisting of a standardized tool and a proprietary questionnaire.

#### 2.2.1 Mental health literacy scale (MHLS-PL)

The study utilized the Mental Health Literacy Scale (MHLS) (42) in the Polish adaptation by Nowicki et al. (MHLS-PL) (43). The Polish version of the MHLS-PL consists of 33 items and is based upon the five key components of mental health knowledge proposed by Jorm et al. (11): Recognition of disorders (1–8 item), Knowledge of risk factors and causes (9–10 item), Knowledge of professional help available (11–13 item), Knowledge of how to seek mental health information (14–17 item) and Attitudes that promote recognition and appropriate help-seeking (18–33 item). Respondents answer using a four- or five-point Likert scale. The total MHL score, as well as the scores for each subscale, are calculated by adding the points, with higher scores indicating a higher level of MHL. Factor analysis in the Polish version of the questionnaire showed the validity of the one-factor and five-factor models (43). Since the individual MHLS-PL subscales' point ranges varied, the raw scores were standardized to a single scale of 0%−100% using the following formula: Normalized score (%) = [(raw score – minimum possible score) / (maximum possible score – minimum possible score)] × 100. This formula was applied to the total MHLS-PL score (range: 33–148) and to each subscale separately. The above allowed for a direct comparison of MHL levels across several dimensions. Cronbach's alpha coefficient for the study group of family nurses was 0.86 for the entire scale and ranged from 0.71 to 0.83 for the individual subscales.

#### 2.2.2 Sociodemographic and occupational variables

The authors developed a questionnaire that included 9 questions about age, gender, place of residence, marital status, education and length of service as a family nurse. In addition, the survey asked if the respondent had been diagnosed with a mental disorder, if a family member or acquaintance/friend of the respondent had been diagnosed with a mental disorder, and if the respondent had ever interacted with a person suffering from a mental disorder in professional work. The possible responses to these questions were “yes”, “no” and “I refuse to answer”.

### 2.3 Ethics approval

The study protocol was approved by the Bioethics Committee at the Medical University of Warsaw (date: 13 October 2025, number: AKBE/291/2025). Respondents' participation in the research was entirely voluntary and anonymous. All of the respondents give their consent to participate in the study. The survey questions were preceded by a page that specified the aim of the study, how to respond the questions and contained an informed consent form. After becoming familiar with the aim of the study, respondents were asked to indicate their readiness to take part in the study by clicking “Yes” or withdraw from the survey by closing the computer browser or clicking “No”. Only respondents who clicked “Yes” were automatically redirected to the survey questionnaire website. Respondents could withdraw from the survey at any time by closing the website.

### 2.4 Statistical analysis

The distribution of categorical variables was presented as numbers and percentages. Continuous variables were summarized as medians with lower and upper quartiles (Q1–Q3) and ranges (min.–max.), as the Shapiro–Wilk test indicated non-normal distribution for most variables. To compare MHLS-PL scores between two independent groups, the Mann–Whitney *U*-test was used. For comparisons involving groups with small sample sizes (*n* < 30 in either group), the exact U statistic is reported. For comparisons with larger sample sizes (*n* ≥ 30 in both groups), the standardized Z statistic (normal approximation) is reported. The association between continuous variables (age, work experience) and MHLS-PL scores was examined using Spearman's rank correlation coefficient (r). To identify factors independently associated with MHLS-PL scores, multiple linear regression was performed. All variables that reached a significance level of *p* < 0.10 in univariable analyses were entered into the initial multivariable model, followed by backward stepwise selection. This approach was chosen for its parsimony in exploratory cross-sectional studies. The results of the linear regression are presented as unstandardized regression coefficients (b) with 95% confidence intervals (CI). The goodness-of-fit of each regression model is described by the coefficient of determination (*R*^2^), which indicates the proportion of variance explained by the included variables. Diagnostic checks for the regression models were performed to verify the underlying assumptions. Linearity and homoscedasticity were evaluated by inspecting scatterplots of standardized residuals against standardized predicted values. The normality of residuals was examined using the Shapiro–Wilk test and Q–Q plots. Multicollinearity was assessed using the Variance Inflation Factor (VIF), with a threshold of VIF < 5 indicating no substantial collinearity (all VIF values in the final models ranged from 1.003 to 1.161). Influential observations were identified using Cook's distance, with values < 1.0 considered acceptable. Autocorrelation of residuals was tested using the Durbin–Watson statistic (values between 1.5 and 2.5 indicating no autocorrelation; observed range across models: 1.595–2.094). Detailed diagnostic results for each regression model are provided in the Supplementary Tables S1, S2. All statistical analyses were performed using IBM SPSS Statistics for Windows, Version 28.0 (Armonk, NY: IBM Corp) and R version 4.4.0 (R: A Language and Environment for Statistical Computing; R Foundation for Statistical Computing, Vienna, Austria). For all tests, a *p*-value < 0.05 was considered statistically significant.

## 3 Results

### 3.1 Characteristics of participants

Table 1 shows the sociodemographic and occupational characteristics of the family nurses under study (*n* = 207). The median age of the study participants was 47 years (Q1–Q3 = 22–76). As many as 97.58% (*n* = 202) of all respondents were women. Given the very small number of male participants, sex-based comparisons should be interpreted with caution. A total of 84.44% (*n* = 181) of all respondents lived in a town or city and 82.61% (*n* = 171) reported that they were in a relationship. In addition, 57.97% (*n* = 120) of all respondents held a master's degree or a doctoral degree. The median length of service in the primary health care was 10 years (Q1–Q3 = 3–27). With respect to experience with persons suffering from mental disorders, 12.56% (*n* = 26) of all respondents reported having a mental disorder themselves, 48.31% (*n* = 100) reported that a family member, friend or acquaintance suffered from a mental disorder and 84.06% (*n* = 174) reported that they interacted with a person who suffered from a mental disorder during their professional work.

**Table 1:** Characteristics of the study group (*n* = 207).

Variable	Statistics
Age (years)^a^	47 (32–55) [22–76]
Gender^b^	
Female	202 (97.58)
Male	5 (2.42)
Place of residence^b^	
Urban	181 (84.44)
Rural	26 (12.56)
Marital status^b^	
Married	171 (82.61)
Single or widow/widower	36 (17.39)
Education^b^	
Bachelor's degree in nursing	87 (42.03)
Master's degree or above	120 (57.97)
Work experience in primary care (years)^a^	10 (3–27) [0.25–50]
Have you been diagnosed with a mental disorder?^b^	
Yes	26 (12.56)
No	181 (87.44)
Have any of your family members or acquaintances/friends been diagnosed with a mental disorder?^b^	
Yes	100 (48.31)
No	107 (51.69)
Did you have contact with someone who had a diagnosed mental disorder in the course of your work?^b^	
Yes	174 (84.06)
No	33 (15.94)

The data is presented as: ^a^Median (Q1–Q3) [min.-max] or ^b^n (%).

### 3.2 Mental health literacy in family nurses and its relationship with selected variables - univariable analyses

Table 2 shows the median and normalized level of mental health literacy assessed through the MHLS-PL scale. The post-normalization MHLS-PL total score is 70.43%. The “Knowledge of risk factors and causes” and “Knowledge of professional help available” subscales had the lowest normalized scores (66.67% each), while the “Recognition of disorders” and “Knowledge of how to seek mental health information” subscales had the highest (75.00% each). The “Attitudes that promote recognition and appropriate help-seeking” subscale had an intermediate score of 68.25%. The highest and lowest subscale scores differed by 8.33 percentage points, which indicates an uneven mental health literacy profile. In particular, competences associated with recognizing mental disorders were discovered to be more developed than knowledge on risk factors and causes of mental disorders.

**Table 2:** Mental health literacy in a group of family nurses.

Mental health literacy	Me (Q1–Q3)	Min.–Max.	Scope		Normalized score (%)
MHLS-PL	114 (108–123)	77–149	33	148	70.43
Subscale 1 - Recognition of disorders	26 (24–28)	8–32	8	32	75
Subscale 2 - Knowledge of risk factors and causes	6 (5–6)	3–8	2	8	66.67
Subscale 3 - Knowledge of professional help available	9 (8–10)	6–12	3	12	66.67
Subscale 4 - Knowledge of how to seek mental health information	16 (14–17)	8–20	4	20	75
Subscale 5 - Attitudes that promote recognition and appropriate help-seeking	59 (55–63)	30–80	16	79	68.25

Me, median; Q1, lower quartile; Q3, upper quartile; Normalized scores were calculated using the median as the measure of central tendency.

Table 3 shows the relationship between mental health literacy and variables under study. Age and length of service as a family nurse were negatively linked with overall mental health literacy and particular MHLS-PL subscales. Furthermore, participants holding a master's degree in nursing or higher, respondents living in urban areas, those who reported having been diagnosed with a mental disorder or having a family member, acquaintance, or friend with such a diagnosis, and those who interacted with people diagnosed with mental disorders in their professional work all had significantly higher levels of mental health literacy. Taking into account the instrument's individual subscales, it was discovered that participants who lived in urban areas, held a master's degree or higher, had been diagnosed with mental disorders or whose family members, acquaintances, or friends had been diagnosed with such disorders, and had interacted with people suffering from mental disorders during their professional work scored significantly higher on the Recognition of disorders subscale. In turn, respondents who stated that their family members, acquaintances, or friends had a mental disorder obtained higher scores on the Knowledge of risk factors and causes subscale. Respondents holding a master's degree in nursing or higher had significantly higher scores on the Knowledge of professional help available subscale, as did those who reported having been diagnosed with a mental disorder, having a family member, acquaintance, or friend with such a diagnosis, or were/are interacting with people diagnosed with mental disorders in their professional work. Moreover, participants holding a master's degree obtained significantly higher scores on the Knowledge of how to seek mental health information subscale. Individuals who had been diagnosed with mental disorders, or whose family members, acquaintances, or friends had been diagnosed with such disorders, and who interacted with people with mental disorders during their professional work obtained significantly higher scores on the Attitudes that promote recognition and appropriate help-seeking subscale.

**Table 3:** Relationship between mental health literacy and variables under study.

Variable	Mental health literacy											
	MHLS-PL	Analysis	Subscale 1	Analysis	Subscale 2	Analysis	Subscale 3	Analysis	Subscale 4	Analysis	Subscale 5	Analysis
	Me (Q1–Q3)		Me (Q1–Q3)		Me (Q1–Q3)		Me (Q1–Q3)		Me (Q1–Q3)		Me (Q1–Q3)	
Age	*r* = −0.337	*p* < 0.001	*r* = −0.230	*p* = 0.001	*r* = −0.259	*p* < 0.001	*r* = −0.173	*p* = 0.013	*r* = −0.202	*p* = 0.003	*r* = −0.285	*p* < 0.001
Gender												
Female	114 (108–122	U = 341 *p* = 0.224	26 (24–28)	U = 387 *p* = 0.386	6 (5–6)	U = 415.5 *p* = 0.510	9 (8–10)	U = 325.5 *p* = 0.181	16 (14–17)	U = 410.5 *p* = 0.486	59 (55–63)	U = 378 *p* = 0.350
Male	123 (123–125)		28 (25–31)		5 (5–6)		10 (10–11)		14 (14–15)		63 (59–67)	
Place of residence												
Rural	110.5 (105–116)	*Z* = −1.933 *p* = 0.055	24.5 (23–16)	*Z* = −2.376 *p* = 0.018	6 (5–6)	*Z* = 0.002 *p* = 0.999	9 (8–10)	*Z* = −1.214 *p* = 0.225	15 (14–16)	*Z* = −0.649 *p* = 0.516	56.5 (55–60)	*Z* = −1.809 *p* = 0.07
Urban	115 (108–123)		26 (24–29)		5 (5–6)		9 (8–11)		16 (14–17)		59 (55–64)	
Relationship status												
Single	114 (108–122)	*Z* = −0.547 *p* = 0.585	26 (24–28)	*Z* = −0.614 *p* = 0.539	5 (5–6)	*Z* = −0.436 *p* = 0.662	6 (8–10)	*Z* = −1.002 *p* = 0.316	16 (14–17)	*Z* = −0.366 *p* = 0.714	59 (55–63)	*Z* = −0.09 *p* = 0.928
In a relationship	117.5 (106.5–126)		26 (24–29)		6 (5–6.5)		9.5 (8–11)		16 (14–17)		58.5 (54–64.5)	
Education												
Bachelor's degree	77 (106–120)	*Z* = −2.252 *p* = 0.024	25 (23–27)	*Z* = −3, 033 *p* = 0.002	5 (5–6)	*Z* = −0.753 *p* = 0.451	9 (8–10)	*Z* = −3.033 *p* = 0.002	8 (13–16)	*Z* = −2.655 *p* = 0.008	58 (54–65)	*Z* = −0.546 *p* = 0.585
Master's degree	116 (109.5–123.5)		26 (24–29)		6 (5–6)		9 (8–11)		16 (14–17)		59.5 (56–63)	
Work experience in primary care	*r* = −0.297	*p* < 0.001	*r* = −0.198	*p* = 0.004	*r* = −0.193	*p* = 0.005	*r* = −0.136	*p* = 0.05	*r* = −0.137	*p* = 0.049	*r* = −0.281	*p* < 0.001
Have you been diagnosed with a mental disorder?												
Yes	123 (115–130)	*Z* = 4.328 *p* < 0.001	28 (26–30)	*Z* = 2.65 *p* = 0.008	6 (5–7)	*Z* = 1.262 *p* = 0.207	10 (8–11)	*Z* = 2.18 *p* = 0.029	16 (15–17)	*Z* = 1.784 *p* = 0.074	64 (60–67)	*Z* = 4.092 *p* < 0.001
No	113 (107–121)		25 (24–28)		5 (5–6)		9 (8–10)		15 (14–16)		59 (54–62)	
Have any of your family members or acquaintances/friends been diagnosed with a mental disorder?												
Yes	120 (112–125)	*Z* = 6.016 *p* < 0.001	27 (25–30)	*Z* = 4.318 *p* < 0.001	6 (5–7)	*Z* = 3.547 *p* < 0.001	9.5 (8–11)	*Z* = 3.575 *p* < 0.001	16 (14–16)	*Z* = 0.568 *p* = 0.570	62 (58–65)	*Z* = 5.328 *p* < 0.001
No	111 (103–119)		25 (23–27)		5 (5–6)		9 (8–10)		15 (13–17)		57 (52–60)	
Did you have contact with someone who had a diagnosed mental disorder in the course of your work?												
Yes	116 (109–123)	*Z* = 2.931 *p* = 0.003	26 (24–28)	*Z* = 3.504 *p* < 0.001	6 (5–6)	*Z* = 1.538 *p* = 0.124	9 (8–11)	*Z* = 2.186 *p* = 0.029	16 (14–17)	*Z* = 1.663 *p* = 0.096	60 (56–64)	*Z* = 2.433 *p* = 0.015
No	111 (102–119)		24 (22–26)		5 (5–6)		9 (8–9)		15 (13–16)		57 (53–60)	

Me, median; Q1, lower quartile; Q3, upper quartile; Group comparisons were performed using the Mann–Whitney U-test. For comparisons with smaller sample sizes (n < 30 in either group), the exact U statistic is reported. For comparisons with larger sample sizes (n ≥ 30 in both groups), the standardized Z statistic (normal approximation) is reported. Correlations were assessed using Spearman's rank correlation coefficient. r, correlation coefficient. Subscale 1: Recognition of disorders; Subscale 2: Knowledge of risk factors and causes; Subscale 3: Knowledge of professional help available; Subscale 4: Knowledge of how to seek mental health information; Subscale 5: Attitudes that promote recognition and appropriate help-seeking.

### 3.3 Multivariable association between mental health literacy and variables analyzed

Diagnostic checks for the multiple linear regression models were performed prior to analysis; a detailed description of the procedures and results is provided in the Statistical analysis section (2.4) and in the Supplementary Tables S1, S2. To assess the stability of the final model, the backward stepwise selection was compared with a forced-entry model including all variables identified as potentially relevant based on clinical and theoretical considerations. The direction and magnitude of the regression coefficients were consistent across both approaches, supporting the robustness of the final model. The forced-entry model also confirmed that the variables retained in the stepwise model were the most consistent correlates of MHL. Table 4 shows the factors independently associated with MHL levels obtained through multiple linear regression. The direction strength of the dependencies revealed in multivariable models were consistent with those obtained in univariable analyses. The following factors were identified as independently associated with the total MHLS-PL score: age, being an urban resident, the occurrence of mental disorders in a family member or acquaintance/friend, and interaction with a person suffering from a mental disorder during the professional work. All variables that were significant in the univariable analyses for the Recognition of disorders subscale, such as age, being an urban resident, holding a master's degree or higher, a diagnosis of a mental disorder in a family member or acquaintance/friend, and professional interaction with people suffering from mental disorders, remained independently associated with this subscale in the multivariable model. With regard to the Knowledge of risk factors and causes subscale, age and a diagnosis of a disorder in a family member, acquaintance or friend were independently associated with this subscale. Having a single status, holding a master's degree or higher, and having a family member, acquaintance, or friend diagnosed with a mental disorder were significantly associated with scores on the Knowledge of professional help available subscale. Factors independently associated with the Knowledge of how to seek mental health information subscale score included holding a master's degree or higher and having shorter length of service as a family nurse. With regard to the Attitudes that promote recognition and appropriate help-seeking subscale, the following factors were independently associated: shorter length of service as a family nurse; a diagnosis of mental disorders in the respondent and a diagnosis of mental disorders in a family member, acquaintance or friend of the respondent. Province size (population above vs. below two million), which was used as a stratification criterion for facility selection, was not included as a variable in the regression analyses, as the primary focus was on individual-level sociodemographic and occupational factors.

**Table 4:** Factors independently associated with mental health literacy (multivariable linear regression).

Variable	*b*	95% CI	*p*	*R* ^2^
MHLS-PL				
Age	−0.186	−0.286; −0.086	< 0.001	0.260
Place of residence (reference category: rural)	4.407	0.310; 8.504	0.035	
Have any of your family members or acquaintances/friends been diagnosed with a mental disorder? (reference category: no)	6.778	3.994; 9.561	< 0.001	
Did you have contact with someone who had a diagnosed mental disorder in the course of your work? (reference category: no)	7.217	3.553; 10.882	< 0.001	
Subscale 1 - recognition of disorders				
Age	−0.033	−0.065; −0.001	0.046	0.214
Place of residence (reference category: rural)	1.550	0.231; 2.868	0.021	
Education (reference category: bachelor's degree)	1.227	0.352; 2.102	0.006	
Have any of your family members or acquaintances/friends been diagnosed with a mental disorder? (reference category: no)	1.654	0.758; 2.550	< 0.001	
Did you have contact with someone who had a diagnosed mental disorder in the course of your work? (reference category: no)	2.382	1.175–3.588	< 0.001	
Subscale 2 - knowledge of risk factors and causes				
Age	−0.017	−0.028; −0.006	0.003	0.091
Have any of your family members or acquaintances/friends been diagnosed with a mental disorder? (reference category: no)	0.409	0.104; 0.714	0.009	
Subscale 3 - Knowledge of professional help available				
Relationship status (reference category: in a relationship)	0.556	0.043; 1.068	0.034	0.132
Education (reference category: bachelor's degree)	0.795	0.392; 1.198	< 0.001	
Have any of your family members or acquaintances/friends been diagnosed with a mental disorder? (reference category: no)	0.862	0.472; 1.252	< 0.001	
Subscale 4 - knowledge of how to seek mental health information				
Education (reference category: bachelor's degree)	0.831	0.211; 1.451	0.009	0.074
Work experience in primary care	−0.034	−0.054; −0.014	0.001	
Subscale 5 - attitudes that promote recognition and appropriate help-seeking				
Work experience in primary care	−0.080	−0.147; −0.012	0.021	0.177
Have you been diagnosed with a mental disorder? (reference category: no)	3.991	1.149; 6.832	0.006	
Have any of your family members or acquaintances/friends been diagnosed with a mental disorder? (reference category: no)	3.965	2.065; 5.864	< 0.001	

^b^regrsesion coefficients; R^2^, coefficient of determination.

## 4 Discussion

This is one of the first studies to employ the validated MHLS-PL instrument to assess the level of MHL among family nurses in Poland. The total MHL score of 70.43% is comparable to the results among nursing students in Poland (43) and nurses in the People's Republic of China (44), indicating a similar level of mental health literacy in these populations. At the same time, there was significant differentiation in the MHL profile, with the highest level obtained in the “Recognition of disorders” and “Knowledge of how to seek mental health information” subscales (75.00% each) and the lowest in the “Knowledge of risk factors and causes” and “Knowledge of professional help available” subscales (66.67% each). The 8.33 percentage point difference between the highest and lowest dimensions shows an uneven distribution of competencies in the field of mental health, with clinical-diagnostic knowledge clearly outweighing etiological and treatment-related knowledge.This profile is consistent with findings from other studies; for instance, nursing students in Poland obtained the lowest scores on the subscale of knowledge about risk factors and causes (43). Hao et al. (45), in turn, demonstrated that nurses employed outside psychiatry had a satisfactory ability to recognize mental disorders, but their knowledge of the etiology and treatment options remained limited. These results indicate that a knowledge gap in terms of the etiological aspect of mental diseases may be a universal problem in this professional group, regardless of geographical or cultural environment. In practice, the above means that the mere ability to identify symptoms cannot be considered sufficient to deliver comprehensive care unless it is supported by in-depth knowledge of risk factors, preventive strategies and clearly defined pathways for referring patients to specialist psychiatric services.

In the multivariable analysis, age was found to be a significant, negative factor associated with the total MHL score and all subscales, with the exception of the “Attitudes that promote recognition and appropriate help-seeking” subscale. A similar association was observed in terms of length of service; longer experience as a family nurse was linked with a considerably lower level of MHL in the “Knowledge of professional help accessible” and “Knowledge of how to seek mental health information” subscales. This unexpected relationship implies that professional experience does not always translate into increased understanding of mental health; rather, it may even degrade over time. A possible interpretation is the lack of regular, updated mental health training under continuing professional development, which may contribute to the persistence of out-of-date or inadequate knowledge. Pietrzak et al. (46) indicated that among nurses working in Polish PHC facilities, the prevalence of anxiety symptoms was 21.71%, depressive symptoms 8%, and irritability 47.43%, which may further impact the ability to acquire new knowledge and maintain a high level of MHL in the long-term perspective.

A master's degree or higher education was significantly associated with higher levels of MHL on the “Recognition of disorders”, “Knowledge of professional help available” and “Knowledge of how to seek mental health information” subscales. The above correlation is consistent with the findings of Wang et al. (44) in a study carried out among nurses in the People's Republic of China, where a higher level of education was one of the most significant predictors of mental health competences. A master's degree, which often covers more advanced content in the social sciences and humanities, can help students gain a better understanding of the complexities of mental health problems, beyond just clinical diagnostic aspects. In addition, academic development can help not only expand theoretical knowledge, but also build the ability to critically analyse health-related information and utilize current scientific knowledge sources.

Being an urban resident was significantly associated with higher MHL scores on the total scale and on the “Recognition of disorders” subscale. This association may be explained by enhanced access to training, conferences and other forms of continuing education in urban facilities, as well as increased exposure to a broader range of clinical cases at PHC facilities located in cities. Alternatively, it could reflect more general disparities between Poland's rural and urban areas in terms of access to mental health services and mental health promotion. However, as these are exploratory hypotheses, further research is needed to confirm the underlying mechanisms. This phenomenon requires further investigation, particularly in the context of planning educational interventions for nurses practicing in rural areas where access to specialized knowledge may be limited.

A subsequent notable observation was the significance of personal and professional experiences related to mental disorders and MHL levels. Having a family member, acquaintance or friend with a recognized mental disorder, as well as professional interaction with patients under psychiatric care, were independently associated with greater levels of MHL. The above result is consistent with current findings, which suggests that direct interaction with persons suffering from mental disorders might enhance knowledge and improve attitudes toward mental health (47), potentially through mechanisms such as reduced stereotyping. This relationship can be interpreted in several ways. Firstly, personal experience with persons suffering from mental disorders might boost one's motivation to learn more about this issue, both intellectually and emotionally. Secondly, contact with persons who have mental disorders may facilitate more positive attitudes toward mental health, which could be reflected in higher scores on the attitude scale. Nevertheless, since stigma was not directly assessed in this study, this interpretation remains speculative and should be examined in future research. Zhamaliyeva et al. (33) in a systematic review with meta-analysis showed that contact-based and multi-component interventions were connected with stronger and more sustained effects in reducing stigma among primary health care professionals. Our results show that a similar mechanism may occur in the case of natural life events, i.e., contact with a person suffering from a mental disease in the local surroundings may constitute a form of “natural intervention” enhancing the level of MHL.

It is worth noting that the respondent's own diagnosis of mental disorders was only significantly associated with higher levels of MHL on the Attitudes that support recognition and appropriate help-seeking subscale. The above may reflect the fact that personal experience associated with a mental disorder has a greater impact on the emotional and attitudinal aspect of mental health literacy, whereas declarative knowledge (for instance, about risk factors or available medical assistance) may be developed by other means. According to the study by Lewandowska et al. (40), a total of 54.8% of all adults in Poland claim to be able to recognize symptoms of mental disorders in themselves and 50.7% in others; however, only 17.2% of respondents believe that sufficient activities are taken in Poland to promote mental health. Our study shows that family nurses, particularly those with personal or familial history with mental disorders, can be a valuable resource in addressing these knowledge deficits at the community level.

The findings of this study should be interpreted within the broader organizational context in which family nurses practice. While individual-level factors such as age, education, and personal experiences were identified as correlates of MHL, these competencies are shaped by and interact with the organizational environment-including training policies, interprofessional collaboration, and institutional support for mental health care (22). Strengthening MHL among family nurses therefore requires not only individual educational interventions but also systemic efforts to create health-literate organizations that enable nurses to apply their knowledge in practice. This perspective aligns with the ongoing health-system reforms that emphasize the reorganization of primary care and the expansion of non-physician roles (25, 48).

The present findings underscore that efforts to strengthen MHL among family nurses should not be confined to continuing education programmes. Rather, they should be part of a broader strategy that addresses organizational health literacy, interprofessional collaboration, and intersectoral partnerships-consistent with the ongoing transformation of primary care toward more integrated, community-oriented models of service delivery.

Beyond the immediate findings, the present study contributes to a broader discussion on the evolving role of health professionals in health-system reform (25). The COVID-19 pandemic has prompted a re-evaluation of how various professional groups can support the reorganization of primary care and community-based services, with particular attention to task-sharing and the expansion of responsibilities for non-physician staff (48). In this context, understanding the mental health literacy of family nurses is not merely an educational issue but also a systemic one, as it relates to the capacity of primary health care to respond to population mental health needs within reformed, community-oriented care models. The professional competencies examined in this study-particularly the ability to recognize mental disorders, understand risk factors, and facilitate appropriate help-seeking-are essential components of the broader health workforce transformation that has been accelerated by the pandemic (49).

The strengths and weaknesses of this study need to be taken into account. Firstly, this is one of the first studies in Poland that comprehensively assesses the level of MHL among family nurses through the validated MHLS-PL instrument. Prior studies have primarily focused on nursing students or the general population, but family nurses, who represent the “first contact point” of the healthcare system, have been under-researched. The study fills a crucial research gap regarding the determinants of MHL in this key professional group in Central and Eastern Europe. Secondly, stratified sampling was utilized, taking into consideration the population of each province and distinguishing between urban and rural areas. The stated approach ensured regional stratification of the sample and allowed for the analysis of discrepancies between urban and rural areas, which is especially significant in light of disparities in access to psychiatric care and mental health promotion in Poland. Thirdly, the applied data quality control procedure, which consists of three stages, considerably improved the reliability of the results. Fourthly, normalizing the subscale scores made it possible not only to assess the total level of MHL, but also to precisely identify areas requiring intervention, especially in terms of knowledge about risk factors and causes of mental disorders. This level of diagnostic detail is of key importance for developing targeted training programs and health policies. Fifthly, a full description of the recruitment procedure, inclusion and exclusion criteria, data quality control, as well as statistical methodology ensured transparency and enabled the study to be replicated in other populations or cultural contexts. Beyond the aforementioned, the employment of the CAWI method reduced the impact of regional barriers and ensured anonymity, which may have enhanced the truthfulness of responses, especially for questions about personal experiences with mental health disorders. Sixthly, the results have direct consequences for clinical practice and health policy, indicating specific areas of deficit and categories of nurses who require further educational training (older nurses, those with longer length of service, and those practicing in rural areas). The identification of personal and family experiences as a factor independently associated with MHL could open up new opportunities for nurses with such experiences to potentially serve as mental health “ambassadors” in future initiatives. However, this role was not evaluated in the present study and should be explored in further research.

Despite these strengths, our study has certain limitations. First, as the study is cross-sectional, it is impossible to make cause-and-effect conclusions concerning the variables under study and the level of MHL. Second, even with stratified sampling, there might not be enough respondents to give a fully representative reflection of the population of family nurses in Poland, which consists of several thousand professionals. This makes it difficult to generalize the findings to the entire population, particularly with regard to subgroup analysis (for instance, nurses with diagnosed mental problems accounted for just 12.56% of the sample). In addition, the sample included only five male nurses (2.42%), reflecting the gender distribution in the Polish nursing profession but limiting the statistical power of any comparisons by sex. In addition, the response rate could not be calculated because invitations were distributed through facility managers rather than sent directly to individual nurses, and the number of nurses who received the invitation could not be determined. Consequently, it is not possible to formally assess non-response bias; however, the final sample of 207 participants includes respondents from all Polish provinces and both urban and rural settings. Third, the recruitment procedure was carried out by primary care facility managers, who were able to distribute invitations among nurses who indicated a greater level of dedication or interest in the research topic under study. Additionally, the selection of PHC facilities was based upon strata defined by population size, but did not take into account other relevant characteristics of the facilities, such as the headcount, ownership structure (public/private), or the degree of urbanization on a more detailed scale, which may have influenced the variation in the MHL level. Fourth, all information, including information about the diagnosis of mental disorders among respondents, family members and friends was based upon self-reports and was not verified against medical records. This could result in either overreporting or underreporting of this data because of stigma, fear of disclosure, or misdiagnosis. In addition, the question about professional interaction with people suffering from mental disorders was of dichotomous nature (yes/no), with no indication of the frequency, nature, or duration of that interaction, preventing a more in-depth analysis of this significant correlate. Fifth, with regard to questions regarding attitudes toward mental health and declarative knowledge, respondents could provide answers that were consistent with social or professional expectations, overestimating the actual level of MHL. This is especially crucial for nurses, who may be under pressure to demonstrate a high level of competence in the field of mental health. Sixth, it should be acknowledged that the MHLS-PL was originally designed for the general community rather than medical professionals. Consequently, the scale does not capture profession-specific competencies such as communication skills or suicide risk assessment, which would require complementary measurement approaches. Nevertheless, the MHLS-PL was selected as a validated instrument for assessing general MHL and has been previously used in primary care populations, including nurses. Seventh, the study relies on self-reported knowledge and attitudes, which is consistent with the cross-sectional survey design and the nature of the constructs measured. Nevertheless, future research could complement these findings with objective assessments of clinical performance. It is therefore unclear if a high level of MHL translates into actual competence in recognizing, managing and supporting patients with mental disorders in day-to-day practice. In light of the above, clinical competence would have to be assessed through additional methods, such as medical simulations, case studies, or supervisor assessments. Eighth, although province size was used as a stratification criterion for sampling, it was not examined as a potential correlate of MHL. Future research could explore how regional characteristics-such as population size, availability of mental health services, and regional training policies-may influence MHL levels among family nurses. Ninth, a limitation of the analytical approach is the use of backward stepwise selection, which may increase the risk of model instability and the exclusion of variables that are theoretically important but not statistically significant in univariable analyses. To mitigate this, we entered all variables with *p* < 0.10 into the initial model and compared the stepwise results with a forced-entry model, which yielded consistent findings. Nevertheless, the results should be interpreted with caution, and future studies with larger samples are recommended to confirm the identified associations. Future research should focus on several key areas. It is critical to conduct longitudinal studies that would allow for the assessment of dynamics in MHL levels over time as well as the effectiveness of educational interventions in this occupational group. At the same time, it is worth employing qualitative methodologies that would allow for a more in-depth understanding of the mechanisms underlying the observed relationships, particularly in terms of how personal experiences impact MHL levels. In addition, comparing family nurses to other PHC personnel groups, such as general practitioners, midwives and social workers, would be useful in identifying areas that require specific intervention. Research on the effectiveness of various training forms, i.e., in person, online and mixed, is especially significant as it can provide practical guidelines to decision-makers in charge of the nursing continuous professional development system.

## 5 Conclusions

This study showed that family nurses in Poland had a median normalized MHL score of 70.43%, with uneven scores across subscales and the greatest knowledge gaps in their understanding of the risk factors and causes of mental disorders. Factors independently associated with the total MHLS-PL score were younger age, urban residence, having a family member or acquaintance with a mental disorder, and professional interaction with individuals with mental disorders. In contrast, educational level (master's degree or higher), shorter length of service, and personal mental health diagnosis were associated only with specific subscales, reflecting domain-specific patterns of MHL. These results also suggest that there may be disparities in the distribution of mental health competences among primary health care nurses, particularly between urban and rural areas and throughout different career stages. However, these findings should be interpreted with caution given the cross-sectional design, the moderate sample size, and the lack of precise response-rate data, which limit the generalisability of the results and preclude causal inferences.

Nevertheless, these findings suggest that specific groups of nurses, particularly those with longer service and older age, may require further educational support. However, improving MHL among family nurses is unlikely to be achieved through individual-level training alone. Sustainable progress will require a broader systems approach that includes: (1) creating supportive organizational environments that enable nurses to apply mental health knowledge in practice; (2) fostering interprofessional collaboration between family nurses, general practitioners, and mental health specialists; (3) building intersectoral partnerships with social services, educational institutions, and community organizations to address mental health holistically; and (4) ensuring adequate health-system resources, including access to specialist referral pathways and continuing professional development opportunities. Such efforts should be embedded within ongoing health-system reforms that support the integration of mental health care into primary care and the continuous professional development of the nursing workforce.

Taking into consideration the key role that family nurses play in primary health care, improving their mental health competences—within a supportive system—can contribute to early recognition of mental health disorders, facilitate timely referrals, and enhance support for patients. This study provides initial empirical insights that can inform the development of targeted educational interventions and human resource strategies, but further longitudinal and implementation research is needed to evaluate the effectiveness of such interventions and to understand how organizational and system-level factors moderate the relationship between nurses' MHL and clinical outcomes.

## Data Availability

The raw data supporting the conclusions of this article will be made available by the authors, without undue reservation.
