# Supplementary material for: Mental health literacy among family nurses in Poland: a cross-sectional study
Source: Front Public Health. 2026 Sep 30;14:1942327. doi: 10.3389/fpubh.2026.1942327 (PMC13661419; doi:10.3389/fpubh.2026.1942327)
Supplement: Supplementary file 1 [file Data_Sheet_1.pdf]

Supplementary Material

Mental Health Literacy Among Family Nurses in Poland: a Cross-Sectional Study

Table S1. Diagnostic statistics for the multiple linear regression models.

Model	Predictors included	VIF range	Durbin-Watson d	Shapiro-Wilk p (residuals)
MHLS-PL	Age; Place of residence; Have any of your family members or acquaintances/friends been diagnosed with a mental disorder?; Did you have contact with someone who had a diagnosed mental disorder in the course of your work?	1.040 - 1.118	1.754	0.173
Subscale 1 - Recognition of disorders	Age; Place of residence; Education; Have any of your family members or acquaintances/friends been diagnosed with a mental disorder?; Did you have contact with someone who had a diagnosed mental disorder in the course of your work?	1.041 - 1.119	1.595	0.859
Subscale 2 - Knowledge of risk factors and causes	Age; Have any of your family members or acquaintances/friends been diagnosed with a mental disorder?	1.105 - 1.105	2.094	0.738
Subscale 3 - Knowledge of professional help available	Relationship status; Education; Have any of your family members or acquaintances/friends been diagnosed with a mental disorder?	1.003 - 1.059	1.858	< 0.001
Subscale 4 - Knowledge of how to seek mental health information	Education; Work experience in primary care	1.004 - 1.004	2.007	0.592
Subscale 5 - Attitudes that promote recognition and appropriate help-seeking	Work experience in primary care; Have you been diagnosed with a mental disorder?; Have any of your family members or acquaintances/friends been diagnosed with a mental disorder?	1.102 - 1.161	2.057	0.116

Note: Although the Shapiro–Wilk test indicated a deviation from normality for Subscale 3 ($p < 0.001$), this is not a critical concern given the sample size ($n = 207$), as multiple linear regression is robust to moderate violations of normality with large samples ($n > 30$). The remaining diagnostic indicators (VIF, Durbin–Watson) were within acceptable ranges.

frontiers
