# Supplementary material for: Mental health literacy among family nurses in Poland: a cross-sectional study
Source: Front Public Health. 2026 Sep 30;14:1942327. doi: 10.3389/fpubh.2026.1942327 (PMC13661419; doi:10.3389/fpubh.2026.1942327)
Supplement: Supplementary file 2 [file Data_Sheet_2.pdf]

Supplementary Material

Mental Health Literacy Among Family Nurses in Poland: a Cross-Sectional Study

Table S2. Variance Inflation Factor (VIF) values for predictors in each regression model.

Model	Predictors	VIF
MHLS-PL	Age	1.114
	Place of residence	1.040
	Have any of your family members or acquaintances/friends been diagnosed with a mental disorder?	1.118
	Did you have contact with someone who had a diagnosed mental disorder in the course of your work?	1.042
Subscale 1 - Recognition of disorders	Age	1.119
	Place of residence	1.041
	Education	1.052
	Have any of your family members or acquaintances/friends been diagnosed with a mental disorder?	1.119
	Did you have contact with someone who had a diagnosed mental disorder in the course of your work?	1.091
Subscale 2 - Knowledge of risk factors and causes	Age	1.105
	Have any of your family members or acquaintances/friends been diagnosed with a mental disorder?	1.105
Subscale 3 - Knowledge of professional help available	Relationship status	1.059
	Education;	1.056
	Have any of your family members or acquaintances/friends been diagnosed with a mental disorder?	1.003
Subscale 4 - Knowledge of how to seek mental health information	Education	1.004
	Work experience in primary care	1.004
Subscale 5 - Attitudes that promote recognition and appropriate help-seeking	Work experience in primary care;	1.102
	Have you been diagnosed with a mental disorder?	1.135
	Have any of your family members or acquaintances/friends been diagnosed with a mental disorder?	1.161
